# Supplementary material for: Evidence for additive and synergistic action of mammalian enhancers during cell fate determination
Source: eLife. 2021 Mar 26;10:e65381. doi: 10.7554/eLife.65381 (PMC8004103; doi:10.7554/eLife.65381)
Supplement: Supplementary file 6. [file elife-65381-supp6.docx]

**Supplementary Table 6 Primer list**

| CD19 Forward | GATGCAGACTCTTATGAGAAC |
| --- | --- |
| CD19 Reverse | TCAGATTTCAGAGTCAGGTG |
| IGJ Forward | TGTTCATGTGAAAGCCCAAG |
| IGJ Reverse | TCGGATGTTTCTCTCCACAA |
| VPREB3 Forward | GGGGACCTTCCTGTCAGTTT |
| VPREB3 Reverse | ACCGTAGTCCCTGATGGTGA |
| CD14 Forward | GATTACATAAACTGTCAGAGGC |
| CD14 Reverse | TCCATGGTCGATAAGTCTTC |
| FCGR1B Forward | CCTTGAGGTGTCATGCGTG |
| FCGR1B Reverse | AAGGCTTTGCCATTTCGATAGT |
| ITGAM Forward | GGGGTCTCCACTAAATATCTC |
| ITGAM Reverse | CTGACCTGATATTGATGCTG |
| GAPDH Forward | TCTCTGCTCCTCCTGTTCGAC |
| GAPDH Reverse | GGCGCCCAATACGACCAAAT |
